# Longitudinal data on parental religious behaviour and beliefs from the Avon Longitudinal Study of Parents and Children (ALSPAC)

**DOI:** 10.12688/wellcomeopenres.15127.2

**Published:** 2019-06-20

**Authors:** Yasmin Iles-Caven, Steven Gregory, Kate Northstone, Jean Golding

**Affiliations:** 1 Bristol Medical School (Public Health Sciences), University of Bristol, Bristol, BS8 2BN, UK

**Keywords:** ALSPAC, religious belief, religious behaviour, longitudinal cohort, atheist, agnostic

## Abstract

There is evidence that, in general, the West is becoming more secular. Religious belief has been shown in some studies to have positive associations with outcomes such as coping with serious illness and other life events and general well-being. In this paper, we describe the data from parents enrolled in the Avon Longitudinal Study of Parents and Children (ALSPAC) on their religious behaviour and beliefs collected on three occasions during the 1990s and early 2000s, that are available for researchers to use in association with other longitudinally collected data on social, biological, genetic and epigenetic features of this culturally largely protestant Christian population. Data were collected antenatally and then subsequently at 5 and 9 years post-delivery from self-completion questionnaires completed by each parent independently.  Strong sex differences (all P<0.001) were noted regarding religious beliefs and behaviour: for example, 49.9% of women stated that they believed in God or some divine being compared with 37% of men. Almost twice as many men (28.6%) than women (14.9%) declared they were atheists. Men were less likely to have stated that they had been helped by a divine presence; to appeal to God if they were in trouble, to attend religious services or obtain help from members of religious groups. Among the 6256 women and 2355 men who answered the questions at all three time points, there was evidence of a slight reduction in professed belief and a slight increase in the proportion stating that they were atheists. Information is available from this resource, which is rich in data on the environment, traumatic incidents, health and genetic background. It can be used for research into various aspects of the antecedents and consequences of religious belief and behaviour.

## Introduction

The general population in the West has become more secular over time ( Chaves, 2017). For example, in the United States, the 2007 Religious Landscape Survey revealed that 92% of Americans believed in God and 56% stated that their faith was very important in their daily lives; a further sweep in 2014 showed that these figures had reduced to 89% and 53% respectively ( The Pew Forum on Religion and Public Life, 2017). In the UK, data from the national censuses of 2001 and 2011 showed that the number of people stating they had no religion increased from 14.8% to 25.1% ( Office of National Statistics, 2012).

Previous research has shown that religious belief is associated with increases in life satisfaction, feelings of hope and self-worth and reductions in depression ( Idler & Kasl, 1997). It has also been shown to be used as a coping mechanism, moderating the effects of stress across the lifespan ( McFadden, 1995; Ness & Wintrob, 1980; Pargament, 1990; Seligman, 1991) although not all studies agree. It has also been reported that some view negative life events as opportunities for spiritual growth ( Ellison, 1991). Cotton and colleagues (2006) confirmed previous research showing that spiritual coping and religious decision-making were positively associated with health outcomes in adolescents. A review by Regnerus (2003) concluded that religiosity produced moderately positive outcomes in adolescents in regard to physical and emotional health, educational attainment, volunteering, involvement in politics and family well-being.

It has been reported that women tend to display higher levels of religiosity (from attitudes to behaviour) than men ( Levin **et al**., 1994; Roth & Kroll, 2007; Spilka *et al*., 2003). Results from a group of US-based national surveys found that older participants were more religious than younger ones ( Levin *et al*., 1994; Taylor *et al*., 1999). The only reduction in religious behaviour reported in the elderly appears to be religious service attendance, perhaps due to ill health and reduced mobility ( Ainlay *et al*., 1992). 

The aim of the present paper is to describe the longitudinal data on religious behaviour and beliefs available from a large longitudinal population birth cohort study—the Avon Longitudinal Study of Parents and Children (ALSPAC)—comprising over 20,000 individuals (women and their partners). 

## Methods

### The ALSPAC sample

All pregnant women resident in the Bristol area of South West England, with expected dates of delivery between 1st April 1991 and 31st December 1992, were invited to enrol in the study. The initial number of pregnancies enrolled was 14,541. Of these initial pregnancies, there was a total of 14,676 fetuses, resulting in 14,062 live births and 13,988 children who were alive by the age of 1 year. Mothers, their partners and the index offspring continue to be followed up via self-completion questionnaires, hands-on clinics and links to routine data collections ( Boyd *et al*., 2013; Fraser *et al*., 2013; Golding & the ALSPAC Study Team, 2004).

At the time of enrolment (and with advice from the ALSPAC Ethics and Law Advisory Committee), it was decided not to enrol the study fathers directly ( Birmingham, 2018). A questionnaire was sent (via the mother) for the partner to complete. The mother could pass this on to him if she wanted her partner to be involved, along with a separate reply-paid envelope. This methodology meant that the study deliberately had no information on whether the mother had invited her partner to take part or not, except on receipt of a completed questionnaire from him. For this reason, reminders could not be sent directly to the partners. In the event, at least one questionnaire was returned by 75% of the partners of the enrolled women.

A major component of the study design was to obtain, from the parents, details of their own personalities, moods and attitudes, including religious behaviour and beliefs, prior to the birth of the index child. The study website contains details of all the data that are available through a fully searchable data dictionary and variable search tool: http://www.bristol.ac.uk/alspac/researchers/our-data/.

### Ethical approval and consent

Ethical approval for the study was obtained from the ALSPAC Ethics and Law Committee and the Local Research Ethics Committees ( Birmingham, 2018). Questionnaires were completed by parents in their own homes – return of a questionnaire to the study offices was interpreted as giving tacit consent to the study. Full details of the approvals obtained are available from the study website ( http://www.bristol.ac.uk/alspac/researchers/research-ethics/). Study members have the right to withdraw their consent for elements of the study or from the study entirely at any time.

### Religious belief questions

The religious behaviour and belief questions were devised specifically for ALSPAC by Ursula King (Emeritus Professor of Theology & Religious Studies, University of Bristol) in discussion with Jean Golding. The same questions were included in self-completion questionnaires to both the mother and her partner during pregnancy and at 5 and 9 years after birth. Table 1 shows the actual wording of the questions used. These questions had been piloted to ensure acceptability prior to their inclusion in questionnaires at the three time points. We suggest that the most important question is the first: ‘Do you believe in God or some divine power?’ The responses: ‘yes; not sure; no’ can be used to divide the responders into: believers, agnostics and atheists respectively.

**Table 1.** Questions asked of the mother and father (antenatally, at 5 and at 9 years after delivery) and their variable names. * Asked at 9 years after delivery only.

	AN	5 years	9 years
*Do you believe in God or in some divine power?* Yes/Not sure/No	*D810* *PB150*	*K6240* *PH6240*	*P4040* *PM4040*
*Do you feel that God (or some divine power) has* *helped you at any time? * Yes/Not sure/No	*D811* *PB151*	*K6241* *PH6241*	*P4041* *PM4041*
*Would you appeal to God for help if you were in* *trouble?* Yes/Not sure/No	*D812* *PB152*	*K6242* *PH6241*	*P4042* *PM4042*
Do you ‘pray’ even if not in trouble? Yes/No *	*-*	*-*	*P4043* *PM4043*
*What sort of religious faith would you say you had?* *(tick only one)* None Church of England Roman Catholic Jehovah’s Witness Christian Science Mormon Other Christian (please describe) Jewish Buddhist Sikh Hindu Muslim Rastafarian Other (please describe)	*D813* *PB153*	*K6243* *PH6243*	*P4044* *PM4044*
*Are you bringing your child up in this faith?* Yes/No *	*-*	*-*	*P4048* *-*
*How long have you had this particular faith?* All my life More than 5 years 3–5 years 1–2 years Less than a year	*D815* *PB154*	*K6246* *PH6246*	*P4047* *PM4047*
*Do you go to a place of worship?* Yes, at least once a week Yes, at least once a month Yes, at least once a year Not at all	*D816* *PB155*	*K6247* *PH6247*	*P4049* *PM4049*
*Do you obtain help and support from leaders of* *your religious group? Yes/No*	*D817* *PB156*	*K6248* *PH6248*	*P4050* *PM4050*
*Do you obtain help and support from other* *members of your religious group?* Yes/No	*D818* *PB157*	*K6249* *PH6249*	*P4051* *PM4051*
*Do you obtain help and support from members of* *other religious groups (please describe)*? Yes/No	*D819* *PB158*	*K6250* *PH6250*	*P4052* *PM4052*

Maternal antenatal questionnaire: “About Yourself” (D variables). Maternal 5-year questionnaire: “Study Mother’s Questionnaire” (K variables). Maternal 9-year questionnaire: “Mother of a 9-Year-Old” (P variables). Paternal antenatal questionnaire: “Partner’s Questionnaire” (PB variables). Paternal 5-year questionnaire: “Study Partner’s Questionnaire” (PH variables). Paternal 9-year questionnaire: “Father of a 9-Year-Old” (PM variables).

As indicated in Table 1, the participants were asked to indicate their faith and were given 12 options, as well as ‘other Christian’ and ‘other’; if they had ticked either box they were asked to describe as text. The text answers have been coded by Y.I.C. See Supplementary Table 3 and Supplementary Table 4 ( Iles-Caven, 2019) for the breakdown of responses and frequencies.

### Timing of the questionnaires

Enrolling women in pregnancy provided a number of technical problems for a study that aimed to enrol all such women who were pregnant and resident in a defined geographic area; by their very nature the women recognised their pregnancies at different stages, the local health services had different criteria as to when pregnancies should receive care, and some women only moved into the area in the second half of pregnancy. The ALSPAC design was for certain questions to be asked at specific gestations, whereas the timing of others was not so crucial. As a result, four questionnaires were designed: one on environmental exposures to be administered as early in pregnancy as possible; one concerning the health and well-being of the woman at 18 weeks and a similar questionnaire at 32 weeks. The remaining questions considered to be important to ask in pregnancy, if at all possible, were included in the questionnaire ‘About Yourself’. If the mother enrolled at gestations <11, 11–14, 15–18, 19–21 or 22–30 weeks, the questionnaire was administered at 14, 23, 26, 28, and 34–37 weeks, respectively. For the 10% of pregnancies that did not enrol until after 30 weeks, the questionnaire was administered at 4 months post-delivery. This questionnaire was 36 pages long and included questions concerning the mother’s medical history, her relationship with her partner, details of her parents, including a measure of her relationship with her mother during childhood, the history of events during her childhood, a social network scale, a perceived social support scale, and a generalised locus of control scale. The religiosity questions comprised three pages and were located between the social support and the locus of control scales at the end of the questionnaire and were answered by 12,351 women.

 The subsequent administration of the religiosity questions to the study mothers occurred when the study child was 5 years 1 month (The Study Mother’s Questionnaire), and 9 years 2 months (Mother of a Nine-Year-Old). The questions were nested within questionnaires, both of which were 48 pages in length, and were answered by 8904 and 7827 women, respectively.

 Questions to the mothers’ partners were included in questionnaires that were sent to the study mother (for forwarding) when she herself was sent those questionnaires containing the religious belief and behaviour questions. These had response rates of 9798, 4484 and 3607 fathers respectively. 

### Description of population

Table 2 presents the distribution of the responding participants in the antenatal period by demographic factors, including their ages, education levels (divided according to the maximum educational achievement in three groups: the lowest <O-level; medium O-level or equivalent; higher <O-level), whether the partner lived with the mother, ethnic background and the sex of the child. It can be seen that the demographic distributions of the women and their partners are similar with the exception of the smaller number of responses from partners who were not living with the mother of their child.

**Table 2.** Proportion (n) of enrolled parents who answered the religion questions in pregnancy by selected sociodemographic factors.

Variable	Mothers, % (n)	Fathers, % (n)
*Age of parents*		
<25	21.2% (2599)	21.3% (2042)
25–34	68.5% (8384)	68.4% (6553)
35+	10.3% (1260)	10.3% (988)
*Parental education level* *		
<O level	28.5% (3304)	26.9% (2436)
O level	35.2% (4089)	34.9% (3154)
>O level	36.3% (4219)	38.2% (3450)
*Partner lives with mother*		
Yes	91.7% (11109)	95.2% (9018)
No	8.3% (1003)	4.8% (456)
*Sex of child*		
Boy	51.5% (6323)	51.5% (4949)
Girl	48.5% (5950)	48.5% (4670)
*Ethnic background*		
White	97.6% (11288)	97.2% (9367)
Non-white	2.4% (273)	2.8% (268)

*Public exams, usually in 5–10 subjects, are normally undertaken at the end of Year 11 (age 16) (although they can be taken at any age). Formerly called ‘O’ (Ordinary) Levels the current equivalent are GCSEs.

Table 3 and Table 4 show the data available from each of the three time points. In order to assess whether there are changes of belief and/or behaviour over time, Supplementary Table 1–Supplementary 2 ( Iles-Caven, 2019) repeat Table 3 and Table 4, but restrict the data to the individuals who responded at all three time points. These data indicate that for both men and women there is a slight reduction in professed belief, and an increase in atheism over time.

**Table 3.** Mother’s beliefs/religion and support at each time point, where data for the questions are available.

Question	Antenatal, n (%)	5 years, n (%)	9 years, n (%)
*Do you believe in God or some divine power?*			
Yes	6160 (49.9%)	4141 (46.5%)	3776 (48.2%)
Not sure	4353 (35.2%)	3018 (33.9%)	2682 (34.3%)
No	1838 (14.9%)	1745 (19.6%)	1369 (17.5%)
*Do you believe that God/divine power has helped you at any time?*			
Yes	4181 (33.9%)	2672 (30.1%)	2566 (32.9%)
Not sure	4672 (37.9%)	3047 (34.3%)	2774 (35.6%)
No	3477 (28.2%)	3152 (35.5%)	2454 (31.5%)
*Would you appeal to God for help if you were in trouble?*			
Yes	5738 (46.6%)	4070 (45.9%)	3578 (45.8%)
Not sure	3861 (31.3%)	2653 (29.9%)	2288 (29.3%)
No	2722 (22.1%)	2146 (24.2%)	1943 (24.9%)
*Mother prays even if not in trouble*			
Yes	-	-	3012 (39.2%)
No	-	-	4677 (60.8%)
*Mother bringing up child in this faith*			
Yes	-	-	5167 (72.0%)
No	-	-	2010 (28.0%)
*Length of time mother has followed her current religion*			
Whole life	8905 (81.8%)	6610 (83.6%)	5667 (80.8%)
>5 years	1472 (13.5%)	1018 (12.9%)	1135 (16.2%)
3–5 years	290 (2.7%)	147 (1.9%)	119 (1.7%)
1–2 years	127 (1.2%)	83 (1.1%)	62 (0.9%)
<1 year	88 (0.8%)	44 (0.6%)	29 (0.4%)
*Frequency mother attends a place of worship*			
At least once a week	885 (7.3%)	886 (10.3%)	927 (12.0%)
At least once a month	836 (6.9%)	849 (9.8%)	723 (9.4%)
At least once a year	3520 (29.2%)	2287 (26.5%)	2235 (28.9%)
Never	6824 (56.6%)	4602 (53.4%)	3838 (49.7%)
*Has the mother received help from:*			
*Leaders in her religious group*			
Yes	897 (7.7%)	645 (7.6%)	738 (10.0%)
No	10735(92.3%)	7789(92.4%)	6620 (90.0%)
*Members of her religious group*			
Yes	1087(9.4%)	856 (10.2%)	921 (12.6%)
No	10465(90.6%)	7499 (89.8%)	6384 (87.4%)
*Members of other religious groups*			
Yes	233(2.1%)	144 (1.8%)	186 (2.6%)
No	11059(97.9%)	7911 (98.2%)	6862 (97.4%)
*Type of religious belief*			
Stated “none”	1981 (16.2%)	1411 (16.1%)	1276 (16.6%)
Church of England	7774 (63.6%)	5532 (63.2%)	4608 (60.1%)
Roman Catholic	972 (7.9%)	670 (7.6%)	583 (7.6%)
Jehovah’s Witness	53 (0.4%)	41 (0.5%)	36 (0.5%)
Christian Scientist	16 (0.1%)	17 (0.2%)	11 (0.1%)
Mormon	30 (0.2%)	22 (0.3%)	16 (0.2%)
Other Christian (please describe) *	851 (7.0%)	797 (9.1%)	425 (5.5%)
Judaism	11 (0.1%)	11 (0.1%)	10 (0.1%)
Buddhist	26 (0.2%)	18 (0.2%)	28 (0.4%)
Sikh	16 (0.1%)	6 (0.1%)	5 (0.1%)
Hindu	22 (0.2%)	13 (0.1%)	6 (0.1%)
Muslim	55 (0.4%)	22 (0.3%)	18 (0.2%)
Rastafarian	5 (0.0%)	<5 (0.0%)	<5 (0.0%)
Other (please describe) *	377 (3.1%)	197 (2.3%)	21 (0.3)

*These descriptors were coded and are described in Supplementary Table S3 ( Iles-Caven, 2019).

**Table 4.** Father’s beliefs/religion and support, where data for the questions are available.

Question	Antenatal	5 years	9 years
*Do you believe in God or some divine power?*			
Yes	3621 (37.0%)	1505 (33.6%)	1275 (35.3%)
Not sure	3376 (34.5%)	1573 (35.1%)	1183 (32.8%)
No	2801 (28.6%)	1406 (31.4%)	1149 (31.9%)
*Do you believe that God/divine power has helped you at any time?*			
Yes	2472 (25.3%)	1031 (23.0%)	876 (24.3%)
Not sure	3158 (32.3%)	1430 (32.0%)	1117 (31.0%)
No	4144 (42.4%)	2013 (45.0%)	1606 (44.6%)
*Would you appeal to God for help if you were in trouble?*			
Yes	3536 (36.2%)	1586 (35.5%)	1248 (34.9%)
Not sure	6288 (27.5%)	1319 (29.5%)	1014 (28.3%)
No	3548 (36.3%)	1566 (35.0%)	1319 (36.8%)
*Father prays even if not in trouble*			
Yes	-	-	902 (25.4%)
No	-	-	2650 (74.6%)
*Father bringing up child in this faith*			
Yes	-	-	2012 (60.7%)
No	-	-	1301 (39.3%)
*Length of time father has followed his current religion*			
Whole life	6671 (79.0%)	3052 (78.3%)	2449 (76.2%)
>5 years	1409 (16.7%)	744 (19.1%)	678 (21.1%)
3–5 years	180 (2.1%)	48 (1.2%)	54 (1.7%)
1–2 years	89 (1.1%)	29 (0.7%)	20 (0.6%)
<1 year	94 (1.1%)	23 (0.6%)	15 (0.5%)
*Frequency father attends a place of worship*			
At least once a week	588 (6.1%)	358 (8.2%)	322 (9.0%)
At least once a month	415 (4.3%)	282 (6.5%)	240 (6.7%)
At least once a year	2515 (26.2%)	987 (22.7%)	952 (26.7%)
Never	6077 (63.3%)	2712 (62.5%)	2049 (57.5%)
*Father receives help from:*			
*Leaders in his religious group*			
Yes	559 (6.0%)	301 (7.1%)	287 (8.2%)
No	8717 (94.0%)	3947 (92.9%)	3198 (91.8%)
*Members of his religious group*			
Yes	642 (7.0%)	335 (7.9%)	327 (9.4%)
No	8544 (93.0%)	3894 (92.1%)	3146 (90.6%)
*Members of other religious groups*			
Yes	144 (1.6%)	65 (1.6%)	55 (1.6%)
No	8944 (98.4%)	4093 (98.4%)	3356 (98.4%)
*Type of religious belief*			
Stated “none”	2523 (26.2%)	1086 (24.9%)	896 (25.6%)
Church of England	5238 (54.3%)	2455 (56.2%)	1849 (52.8%)
Roman Catholic	699 (7.3%)	314 (7.2%)	274 (7.8%)
Jehovah’s Witness	32 (0.3%)	21 (0.5%)	12 (0.3%)
Christian Scientist	13 (0.1%)	7 (0.2%)	<5 (0.1%)
Mormon	18 (0.2%)	13 (0.3%)	5 (0.1%)
Other Christian (please describe) *	591 (6.2%)	374 (8.6%)	166 (4.7%)
Judaism	7 (0.1%)	5 (0.1%)	<5 (0.1%)
Buddhist	29 (0.3%)	11 (0.3%)	21 (0.6%)
Sikh	18 (0.2%)	<5 (0.1%)	<5 (0.1%)
Hindu	19 (0.2%)	<5 (0.1%)	<5 (0.1%)
Muslim	59 (0.6%)	16 (0.4%)	10 (0.3%)
Rastafarian	5 (0.1%)	<5 (0.0%)	<5 (0.1%)
Other (please describe) *	381 (4.0%)	91 (2.1%)	6 (0.2%)

*These descriptors were coded and are described in Supplementary Table S4 ( Iles-Caven, 2019).

When the children were 9 years of age, 72.3% of mothers claimed they were bringing up their child in their faith ( Table 5). This includes those that stated they had no religion (31.2%).

**Table 5.** Maternal response to the question “Are you bringing your child up in this faith?” asked when the child was 9 years old.

Mother’s faith	Mother bring up child in this faith		Total
	Yes	No	
None	269 (31.2%)	594 (68.8%)	863 (100.0%)
Church of England	3699 (82.2%)	799 (17.8%)	4498 (100.0%)
Roman Catholic	405 (69.2%)	180 (30.8%)	585 (100.0%)
Jehovah’s Witness	32 (84.2%)	6 (15.8%)	38 (100.0%)
Methodist, Baptist or other Christian	604 (70.0%)	259 (30.0%)	863 (100.0%)
Buddhist	7 (25.9%)	20 (74.1%)	27 (100.0%)
Other	131 (53.9%)	112 (46.1%)	243 (100.0%)
**All responses**	**5147 (72.3%)**	**1970 (27.7%)**	**7117 (100.0%)**

## Strengths and limitations of the data

The primary strength of this data set is the size of the sample of participants and the fact that responders comprise a general population of over 20,000 men and women with no restrictions on their selection other than that they were initially expecting a baby. They were roughly representative of the local Avon population in terms of socioeconomic status (slightly more likely to be owner-occupiers, own a car and be married, and live in over-crowded circumstances) but less likely to be non-White ( Fraser *et al*., 2013). The initial religious behaviour and belief questions were asked during pregnancy and responses at that point were not influenced by the birth nor future characteristics of the child. Identical questions were asked 5 and 9 years after the birth of the child. A major advantage of the data is that it can be linked to information already collected on the individuals including: (a) characteristics of their parents, (b) their own childhoods including their health, well-being and traumatic events, (c) their social and educational background, (d) their personality, attitudes and behaviour, (e) their interactions with their children, (f) and future outcomes. In addition, the data can be linked to characteristics of the child such as development, health and well-being.

One limitation of this data is that extrinsic and intrinsic religiosity were not measured directly which prevents more complex analyses. Extrinsic individuals are more likely to exploit religion, e.g. to provide security and solace, for social reasons, status and self-justification. Intrinsic individuals aim to live their life according to the tenets of that religion and exhibit behaviours consistent with those tenets ( Allport & Ross, 1967).

 Other limitations concern the reduction in the numbers of men answering the questionnaires; this was largely due to the fact that the mother was seen as the centre of the enrolment, and consequently there was no direct contact with the study fathers. A further limitation is the lack of diversity, because at the time of enrolment, the county of Avon was mainly Caucasian, and there were too few non-white participants (<6%) to enable analysis by ethnic background.

## Data availability 

### Underlying data

ALSPAC data access is through a system of managed open access. The steps below highlight how to apply for access to the data included in this paper and all other ALSPAC data. Note that Table 1 in this paper gives the variable numbers for the religion data. Please read the ALSPAC access policy ( http://www.bristol.ac.uk/media-library/sites/alspac/documents/researchers/data-access/ALSPAC_Access_Policy.pdf) which describes the process of accessing the data and biological samples in detail, and outlines the costs associated with doing so.

1. You may also find it useful to browse our fully searchable research proposals database ( https://proposals.epi.bristol.ac.uk/), which lists all research projects that have been approved since April 2011.
2. Please submit your research proposal ( https://proposals.epi.bristol.ac.uk/) for consideration by the ALSPAC Executive Committee using the online process. You will receive a response within 10 working days to advise you whether your proposal has been approved.
  If you have any questions about accessing data, please email: alspac-data@bristol.ac.uk (data) or bbl-info@bristol.ac.uk (samples).
  The ALSPAC data management plan ( http://www.bristol.ac.uk/media-library/sites/alspac/documents/researchers/data-access/alspac-data-management-plan.pdf) describes in detail the policy regarding data sharing, which is through a system of managed open access.

### Extended data

Open Science Framework: Longitudinal data on parental religious behaviour and beliefs from the Avon Longitudinal Study of Parents and Children (ALSPAC). https://doi.org/10.17605/OSF.IO/KX2EN ( Iles-Caven, 2019). The following tables are included in Supplementary Tables.pdf:

- Supplementary Table 1. Maternal: where data for each question are available for all time points
- Supplementary Table 2. Paternal: where data for each question are available for all time points
- Supplementary Table 3. Coding of the ‘Other Christian’ and ‘Other’ – Mothers
- Supplementary Table 4. Coding of the ‘Other Christian’ and ‘Other’ – Partners

Extended data are available under the terms of the Creative Commons Zero “No rights reserved” data waiver (CC0 1.0 Public domain dedication).
