# Supplementary material for: Longitudinal data on parental religious behaviour and beliefs from the Avon Longitudinal Study of Parents and Children (ALSPAC)
Source: Wellcome Open Res. 2019 Jun 20;4:38. Originally published 2019 Feb 21. [Version 2] doi: 10.12688/wellcomeopenres.15127.2 (PMC6589926; doi:10.12688/wellcomeopenres.15127.2)
Supplement: Peer review history

# Peer review: Reviewer response for version 2

**DOI:** 10.21956/wellcomeopenres.16746.r35802

**Published:** 2019-06-21

**Authors:** Philip Wilson

**Affiliations:** 1 Centre for Rural Health, Centre for Health Sciences, University of Aberdeen, Inverness, UK

The authors have addressed my concerns about the original draft of this manuscript very well and I am now happy to approve the paper.

I confirm that I have read this submission and believe that I have an appropriate level of expertise to confirm that it is of an acceptable scientific standard.

# Peer review: Reviewer response for version 1

**DOI:** 10.21956/wellcomeopenres.16505.r35459

**Published:** 2019-05-17

**Authors:** Philip Wilson

**Affiliations:** 1 Centre for Rural Health, Centre for Health Sciences, University of Aberdeen, Inverness, UK

Thank you for the opportunity to review this useful paper based on parental responses to ALSPAC questionnaires in the antenatal period and at child age five and nine years. The findings are in many respects unsurprising: greater levels of religious belief and adherence among women than men and modestly reducing levels of religious affiliation over time. The purpose of the paper is to highlight the availability of the data so the analysis is relatively straightforward. It would nevertheless have been good to see, for example, an illustrative regression analysis comparing those who lose their religious belief with those who maintain it.

 I have a few concerns about the tone in places: there seems to be an assumption that the often-reported association between religious belief and better health outcomes is causal. For example, in the abstract there is a statement “Religious belief has been shown in some studies to positively impact on outcomes such as coping with serious illness and other life events and general well-being”. While this may be true, in the absence of experimental studies it is equally plausible that confounders account for the association so it would probably be better to use the word ‘association’ rather than ‘impact’.

 The introduction is good but it is something of an exaggeration to state that there have been few population studies of religious affiliation: there have been a fairly large number of cross sectional studies in several countries. The particularly valuable feature of the ALSPAC data is that they are longitudinal.

 The British Social Attitudes Survey in 2016 gave a figure of 53% for lack of religious affiliation, significantly higher than that found in the 2011 census and substantially higher than that found in the BSAS in 1983 (31%). This large, and possibly accelerating, reduction in religious affiliation makes it particularly important to place the ALSPAC data in its chronological context.

 The limitations of the data are well discussed. The mode of recruitment and selective attrition of fathers certainly presents some difficulties in interpretation. One additional concern may be in interpretation of the question “Are you bringing your child up in this faith?”. It would be useful to know whether this answered in the affirmative by significant numbers of those professing atheism.

 Minor points:

- There is some repetition in the abstract (sentences about data availability).
- The abstract should make it clear that the data were collected during the 1990s and early 2000s.
- There are not many Buddhists in the sample but they present a problem in interpreting the Religious Belief questions *“‘Do you believe in God or some divine power?’ The responses: ‘yes; not sure; no’ can be used to divide the responders into: believers, agnostics and atheists respectively.*” Buddhists are religious but not theists.
- ‘O-level’ probably needs clarification for an international readership.

I confirm that I have read this submission and believe that I have an appropriate level of expertise to confirm that it is of an acceptable scientific standard, however I have significant reservations, as outlined above.

# Peer review: Reviewer response for version 1

**DOI:** 10.21956/wellcomeopenres.16505.r34946

**Published:** 2019-03-12

**Authors:** Ralph W. Hood Jr.

**Affiliations:** 1 Department of Psychology, University of Tennessee at Chattanooga, Chattanooga, TN, USA

This is a useful study framed with the larger context of the increasing secularization of western cultures, especially in the United States and United Kingdom belief in God continues to decline and more people identify as spiritual independent of specific religious beliefs and participation. Insofar as previous research has established that religiosity is associated with many positive physical and psychological health outcomes, as well as general life satisfaction, decline in religiosity over time may suggest issue of concern with regards to the largely positive effects of religiosity that may be less available to individuals over their lifespan with the increasing secularization of cultures.

 This study provides longitudinal data tracking primarily birth mothers and their surviving children at three time periods, antenatal, 5 and 9 years. This is a large longitudinal birth cohort study of birth mothers and their children the Avon Longitudinal Study of Parents and Children (ALSPAC) ( http://www.bristol.ac.uk/alspac/). In addition more limited data are collected from male partners if they responded to a questionnaire sent via the birth mother. Of particular value are religious measures that allow the tracking of change in beliefs and practices over time.

 Among the more significant findings are that mothers tend to remain committed to religion over time although just under half believe in God or some divine power and another third are not sure. Males are almost equally distributed across the three options, a third each believing in God or some divine power, a third uncertain, and a third confident in their denial of either a God or some divine power. Not surprisingly, belief that one has been helped by God or some divine power follows the percentages associated with belief, uncertainty, and denial of God or some divine power. While slightly over 70% of mothers (as opposed to 60% for fathers) are bringing up their child in their religion, neither mothers nor fathers are likely to pray and not surprisingly unlikely to appeal to God or a divine power in times of trouble. Religious attendance is infrequent among both mothers and fathers with “never” or “at least once a year” the most common choices, for both mothers and fathers. Finally, it is not surprising that neither mothers nor fathers report receiving help from leaders in their religious group. Thus, the overall picture that emerges from these longitudinal data are stable patterns that change little over time and indicate that religion if seen as significant, is more so for mothers than fathers, but overall whether belief, assistance in time of trouble, or prayer, religion appears to play a significant role for less than half the mothers in the ALSPAC study.

 Additional data is available that will allow interested investigators to seek correlates of religious beliefs or practices. Also, given the large sample size one might be able to shed light on why it is that some individuals maintain beliefs and practices that others abandon. There appears to be for most questions (especially with mothers) a consistent trajectory with a distinct dip at 5 years and then a return at 9 years to the previous antenatal high.

 Limitations of this study are properly noted by the authors and include failure to directly measure religious motivations (extrinsic/intrinsic), the lack of a diverse religious sample (over 60% Church of England) and the concomitant lack of other diversity (such as ethnicity) and the limited response from male partners as opposed to birth mothers. Yet despite these criticisms the ALSPAC study provides useful longitudinal data in an area where most research is cross-sectional.

I confirm that I have read this submission and believe that I have an appropriate level of expertise to confirm that it is of an acceptable scientific standard.
